# A Longitudinal Study of the Bidirectional Temporal Dynamics Between Body Mass Index and Biological Aging

**DOI:** 10.1002/jcsm.13824

**Published:** 2025-05-08

**Authors:** Peggy Ler, Jonathan K. L. Mak, Chandra A. Reynolds, Alexander Ploner, Nancy L. Pedersen, Juulia Jylhävä, Anna K. Dahl Aslan, Deborah Finkel, Ida K. Karlsson

**Affiliations:** ^1^ Department of Medical Epidemiology and Biostatistics Karolinska Institutet Solna Sweden; ^2^ Department of Pharmacology and Pharmacy Li Ka Shing Faculty of Medicine The University of Hong Kong Hong Kong SAR China; ^3^ Institute for Behavioral Genetics and Department of Psychology and Neuroscience University of Colorado Boulder Boulder Colorado USA; ^4^ Faculty of Medicine and Health Technology and Gerontology Research Center University of Tampere Tampere Finland; ^5^ Tampere Institute for Advanced Study Tampere Finland; ^6^ School of Health Sciences University of Skövde Skövde Sweden; ^7^ Center for Economic and Social Research University of Southern California Los Angeles California USA; ^8^ Institute for Gerontology Jönköping University Jönköping Sweden

**Keywords:** biological aging, body mass index, dual change score, frailty index, obesity, trajectory

## Abstract

**Background:**

Obesity and aging share biological processes, but their relationship remains unclear, especially in late life. Understanding how body mass index (BMI) and biological aging influence each other can guide strategies to reduce age‐ and obesity‐related health risks. We examined the bidirectional, longitudinal association between changes in BMI and biological aging, measured by frailty index (FI) and functional aging index (FAI), across late life.

**Methods:**

This longitudinal cohort study used data from the Swedish Twin Registry substudies, GENDER, OCTO‐Twin and SATSA, collected via in‐person assessments from 1986 to 2014 at 2‐ to 4‐year intervals. We analysed 6216–6512 evaluations from 1902 to 1976 Swedish twins. Dual change score models were applied to assess the bidirectional, longitudinal association between BMI and FI or FAI from ages 60.0–91.9. FI measured physiological aging, while FAI assessed functional aging through a composite score of functional abilities.

**Results:**

At first measurement, mean age was 74 ± 8, and 41% were males. BMI–FI relationship was bidirectional (*p* value ≤ 0.001): Higher BMI predicted a greater increase in FI over time (coupling effect [*γ*] = 0.86, 95% confidence interval [CI] = 0.65–1.06, *p* value ≤ 0.001), and higher FI predicted steeper decline in BMI (*γ* = −0.04, 95% CI = −0.05 to −0.03, *p* value ≤ 0.001). When including coupling from FI, BMI showed a nonlinear trajectory with a mean intercept of 26.32 kg/m^2^ (95% CI = 25.76–26.88), declining more rapidly after age 75. When including BMI coupling, FI increased from a mean intercept of 7.91% (95% CI = 6.41–9.42), with steeper growth from ages 60–75. BMI–FAI relationship was unidirectional (*p* value ≤ 0.001): Higher FAI predicted a steeper BMI decline (*γ* = −0.02, 95% CI = −0.02 to −0.01, *p* value ≤ 0.001). By including FAI coupling, BMI had a mean intercept of 26.10 kg/m^2^ (95% CI = 25.47–26.74), declining rapidly after age 75. FAI increased exponentially from a mean intercept of 36.49 (95% CI = 34.54–38.43).

**Conclusions:**

Higher BMI predicted a steeper increase in FI, substantiating the hypothesis that obesity accelerates biological aging. Higher biological aging, measured as FI and FAI, drove a steeper BMI decline in late life, signalling that late‐life weight loss may result from accelerated aging. Higher BMI may accelerate aspects of the aging process, and the aging process, in turn, accelerates late‐life BMI decline, necessitating an integrated approach to manage both obesity and unintentional weight loss among older adults.

## 1 Background

Two significant trends have emerged in recent decades: the exponential growth in obesity prevalence and an aging population [1, 2]. As obesity prevalence increases across life stages, increased mid‐life and late‐life obesity rates are projected to rise [3, 4]. High body mass index (BMI) and aging contribute to chronic illnesses, prompting a focus on understanding their connection to mitigate related health complications [5, 6].

Obesity and aging share pathophysiological features like metabolic dysfunction, increased inflammation and insulin resistance, predisposing individuals to similar chronic diseases [5, 6]. The overlapping pathophysiological characteristics and comorbidities have led to the hypothesis that obesity accelerates aging [5, 6]. This hypothesis is supported by existing research, which found obesity associated with increased biological aging, a measure beyond chronological age to capture interindividual differences during aging [5, 6]. Specifically, obesity was associated with an increased risk of frailty, a measure of physiological vulnerability and biological aging [7, 8].

However, the nature of the association between BMI and biological aging remains uncertain, as most studies relied on single‐time‐point data, limiting the possibility of concluding the direction of the effects [7]. Biological age (BA) and BMI can fluctuate over the life course, and changes may result from diverse causes influenced by genetic, environmental and social factors [9, 10, 11]. During aging, BMI may increase due to innate processes such as a decline in sex hormones or total energy expenditure [12, 13]. However, BMI may also decline due to unintentional weight loss due to prodromal phases of diseases or the escalating severity of underlying illnesses [14]. Therefore, the ‘obesity paradox’, where higher BMI appears protective in late life, may be explained by reverse causality [15]. Examining longitudinal changes in BMI and biological aging is essential to fully understand how obesity and aging affect each other and the direction of their association.

Accordingly, our study aimed to investigate the direction of the association between BMI trajectories and BA trajectories while considering dynamic changes during aging. We used BA measured by the frailty index (FI) [16] and functional aging index (FAI) [17], corresponding to assessments of aging multidimensionally and at physiological and functional levels, respectively. Using multiple assessments of BMI, FI and FAI from mid to late life, we examined the chronological sequence of changes. Thereby, we could test whether a change in BMI predicts a change in biological aging or if a change in biological aging predicts a change in BMI.

## 2 Methods

We used data from longitudinal substudies in the Swedish Twin Registry, GENDER [18], OCTO‐Twin [19] and SATSA [20]. Participants completed in‐person testing (IPT) conducted by trained healthcare professionals. GENDER, OCTO‐Twin and SATSA comprised 498, 702 and 859 individuals, respectively, completing at least one IPT. These studies implemented three, five and 10 waves of IPTs every 2–4 years from 1986 to 2014 (Figure S1). The participants' ages ranged from 70 to 88 years in GENDER, 80 to 102 in OCTO‐Twin and 50 to 100 in SATSA. All participants provided informed consent, and the studies were approved by the Ethics Committee in Stockholm.

### 2.1 BMI and BA Assessments

Weight and height were measured during each IPT. BMI was computed by dividing weight by the square of height.

FI is a multidimensional measure of biological aging, expressed as the proportion of health deficits [16]. It represents the percentage of health deficits present out of the total number of deficits considered. FI was computed from 42 self‐reported health deficits in GENDER and SATSA and 41 from OCTO‐Twin, detailed elsewhere (Table S1) [21]. A higher value of FI corresponds to a higher BA, with deficits including symptoms and diseases, such as cardiovascular and respiratory diseases, mental health and the ability to manage activities of daily living.

FAI is a physiological and functional measure of biological aging that combines gait speed, grip strength, peak expiratory flow and self‐reported subjective sensory ability (vision and hearing) [17]. It is a composite score standardized to a mean of 50 with a standard deviation of 10 using values from IPT1. A higher FAI corresponds to worse physical functioning.

### 2.2 Covariates

We included sex (coded as 0 for male and 1 for female), smoking status (coded as 0 for those who never smoked and 1 for those who ever smoked) and study (contrast coded as 0.5 for GENDER, 1 for OCTO‐Twin and −1.5 for SATSA) as covariates. We applied customized contrast coding for the covariate study to account for differences in age distribution and sample size, allowing its effects to be modelled with a single parameter.

### 2.3 Statistical Analyses

We employed bivariate dual change score models (DCSMs) to examine the bidirectional association between levels and changes in BMI and BA, assessed using either FI or FAI in late life [22]. From this point forward, FI and FAI will collectively be called BA. The analytical workflow (Figure S2) began by using chronological age as the underlying time scale, where data were divided into 2‐year age bins. We included participants with at least one BMI and BA assessment (BA) and excluded measurements taken before age 60 and after age 91.9 to ensure a minimum of 100 BMI and BA assessments in each age bin.

Next, we modelled the mean age trajectory and individual variations of change in BMI and each BA separately through univariate DCSMs. Univariate DCSM includes a constant linear change (*α*) set to one, estimates of the intercepts (BMI_0_ or BA_0_), slopes (BMI_slope_ or BA_slope_) and a proportional change (*β*_BMI_ or *β*_BA_), which may or may not be included. The change in BMI over 2 years at age (ΔBMI_*t*_) with proportional change is represented as

$${\text{ΔBMI}}_{t}=\alpha \times {\mathrm{BMI}}_{\text{slope}}+\beta_{\mathrm{BMI}}\times \left({\mathrm{BMI}}_{t-1}\right),$$

where the slope represents the linear change and *β* represents the nonlinear change by multiplying *β* with measures from the previous time point. We compared two models: (1) a model without a proportional change coefficient, where the change between measurements is a function of the slope only, thus assuming a linear trajectory, and (2) a model with a proportional change coefficient, where the change between measurements is a function of both the slope and the variable's level at the previous measurement multiplied by the proportional change coefficient. The first model assumes that change occurs at a constant linear rate, while the second model allows for feedback effects from prior variable levels leading to exponential trajectories [22]. The BMI and BA trajectories were determined by selecting the best‐fitting univariate DCSM with the lowest Akaike information criteria (AIC) and log‐likelihood ratios (−2LL) and using significance tests by comparing nested models using likelihood ratio tests (LRTs).

**FIGURE 1:**
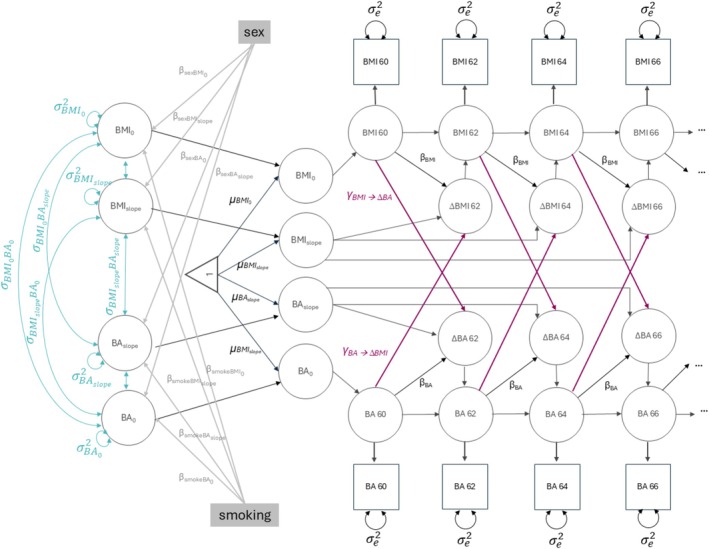
Path diagram of a bivariate dual change score model of body mass index (BMI) and biological age (BA). BMI 60, BMI 62, BMI 64 and BMI 66 denote BMI levels at the first to fourth time point, which corresponds to BMI levels at ages 60–61.9, 62–63.9, 64–65.9 and 66–67.9, respectively. BA 60 to BA 66 denote BA levels from the first to the fourth time point corresponding to BA levels at ages 60–61.9, 62–63.9, 64–65.9 and 66–67.9, respectively. The figure illustrates levels of BMI and BA and the change of BMI and BA from ages 60 to 67.9, while the full model includes levels and changes from ages 60.0–91.9 years. BMI_0_, BA_0_, BMI_slope_ and BA_slope_ represent intercepts and slopes of BMI and BA. The estimated mean levels are denoted by *μ*, variance by *σ* ^2^ and covariance by *σ*. Change in BMI and BA from one time point to the next is represented by ΔBMI 62–66 and ΔBA 62–66 and is made up of respective slopes together with *β*_BMI_ and *β*_BA_, which represent the proportional changes that link change in BMI and BA to the level at the previous time point. *γ*_BMI⟶ΔBA_ represents the coupling parameter that links BA change to BMI level at the previous time point, and *γ*_BA⟶ΔBMI_ represents the coupling parameter that links BA change to BMI level at the previous time point. The models were adjusted for sex, smoking status, study and twin relatedness, although only adjustments for sex and study are represented in the path diagrams. *β*_sexBMI0_ and *β*_smokeBMIslope_ denote coefficients of sex and smoking on BMI intercepts and slopes, and *β*_sexBA0_ and *β*_smokeBAslope_ denote coefficients of sex and smoking on BA intercepts and slopes. BA—biological age, BMI—body mass index.

After determining the best‐fitting univariate DCSM for BMI and BA, we incorporated the models into bivariate DCSMs to assess coupling parameters (*γ*) that link BMI levels to changes in BA and BA levels to changes in BMI to evaluate the direction of the association (Figure 1). The coupling parameters estimate how BMI levels at time *t* − 1 associate with the change in BA (*γ*_BMI⟶ΔBA_) from *t* − 1 to *t*, or vice versa, how BA levels at time *t* − 1 associate with change in BMI (*γ*_BA⟶ΔBMI_), over and above correlations between intercept levels and linear slopes. To examine bidirectional and unidirectional associations between BMI and BA, we compared four model specifications: (1) a fully bidirectional model where both coupling parameters *γ*_BMI⟶ΔBA_ and *γ*_BA⟶ΔBMI_ were estimated, (2) a unidirectional model where only *γ*_BMI⟶ΔBA_ was estimated (while *γ*_BA⟶ΔBMI_ was fixed to zero), (3) a unidirectional model where only *γ*_BA⟶ΔBMI_ was estimated (while *γ*_BMI⟶ΔBA_ was fixed to zero) and (4) a model where both coupling parameters were fixed to zero thus implying no bivariate association. The equation estimating the change in BMI incorporating coupling parameters is represented as

$${\text{ΔBMI}}_{t}=\alpha \times {\mathrm{BMI}}_{\text{slope}}+\beta_{\mathrm{BMI}}\times \left({\mathrm{BMI}}_{t-1}\right)+\gamma_{\mathrm{BA}⟶\text{ΔBMI}}\;\times {\mathrm{BA}}_{t-1}.$$

Similarly, changes in BA can be represented as

$${\mathrm{\Delta BA}}_{t}=\alpha \times {\mathrm{BA}}_{\text{slope}}+\beta_{\mathrm{BA}}\times \left({\mathrm{BA}}_{t-1}\right)+\gamma_{\mathrm{BMI}⟶\mathrm{\Delta BA}}\times {\mathrm{BMI}}_{t-1}.$$

The direction of the association was determined by comparing the models that included coupling in both directions to those that included coupling in either direction or none at all, using AIC, −2LL and LRTs of nested models.

All models were adjusted for sex, smoking status and study on the intercepts and slopes, thus adjusting for the first level and constant linear change of BMI and BA. The models were also adjusted for twin relatedness by computing variances and covariances of the intercepts and linear slopes at the twin‐pair levels. Additionally, the models estimate covariances between their respective intercepts and slopes, variances of the intercepts and slopes and residual variances. All analyses were performed with R Version 4.2.3 and the OpenMx package (Version 2.21.11) [23]. See ‘https://peggyler.github.io/DCSM_OpenMx_code’ for the OpenMx codes used for DCSMs.

## 3 Results

Table 1 describes the characteristics of the participants. Analyses with FI included 1976 participants with at least one BMI and/or FI assessment, out of whom 1291 had at least three evaluations. Analyses with FAI included 1902 participants with at least one BMI and/or FAI assessment, out of whom 1207 participants had at least three evaluations (Table S2).

**TABLE 1:** Characteristics of the analytical sample at first measurement.

	FI sample	FAI sample
N. participants	1976	1902
N. observations	6512	6216
Age in years, mean (SD)	74.3 (8.3)	73.9 (8.2)
BMI in kg/m^2^, mean (SD)	25.6 (4.0)	25.6 (4.0)
FAI, mean (SD)		48.0 (11.7)
FI in %, mean (SD)	15.7 (10.3)	
Males, *n* (%)	804 (40.7)	776 (40.8)
Females, *n* (%)	1172 (59.3)	1126 (59.2)
Never smoked, *n* (%)	1026 (51.9)	980 (51.5)
Ever smoked, *n* (%)	950 (48.1)	922 (48.5)
Study cohort		
GENDER	498 (25.2)	493 (25.9)
OCTO‐Twin	660 (33.4)	591 (31.1)
SATSA	818 (41.4)	818 (43.0)

*Note:* Mean and standard deviation of the first measurement are reported for continuous variables. Numbers and percentages of the total number of participants included in the study, and thus all participants who had at least one BMI, FI or FAI assessment, are reported for categorical variables.

Abbreviations: BMI—body mass index, FAI—functional aging index, FI—frailty index, *n*—number, SD—standard deviation.

### 3.1 Univariate Trajectories of BMI and BA

Table S3 presents the fit statistics guiding the selection of the univariate DCSM. For BMI, a model with a proportional change parameter was preferred over one without or a linear change model (*p* value based on LRT ≤ 0.001), FI (*p* value ≤ 0.001) and FAI (*p* value ≤ 0.001). Change in BMI was driven by a negative linear slope, compensated by positive proportional effects (Table S4). This resulted in BMI trajectories showing declining trends from 60 to 91.9 years (Figures S3 and S4). Changes in FI and FAI were driven by a negative linear slope and a positive proportional change parameter, resulting in FI and FAI trajectories showing an exponential increase over age (Tables S5 and S6 and Figures S5 and S6).

### 3.2 Bivariate Associations Between Changes in BMI and BA

The fit statistics demonstrating the selection of bivariate DCSMs are presented in Tables S7 and S8.

**FIGURE 2:**
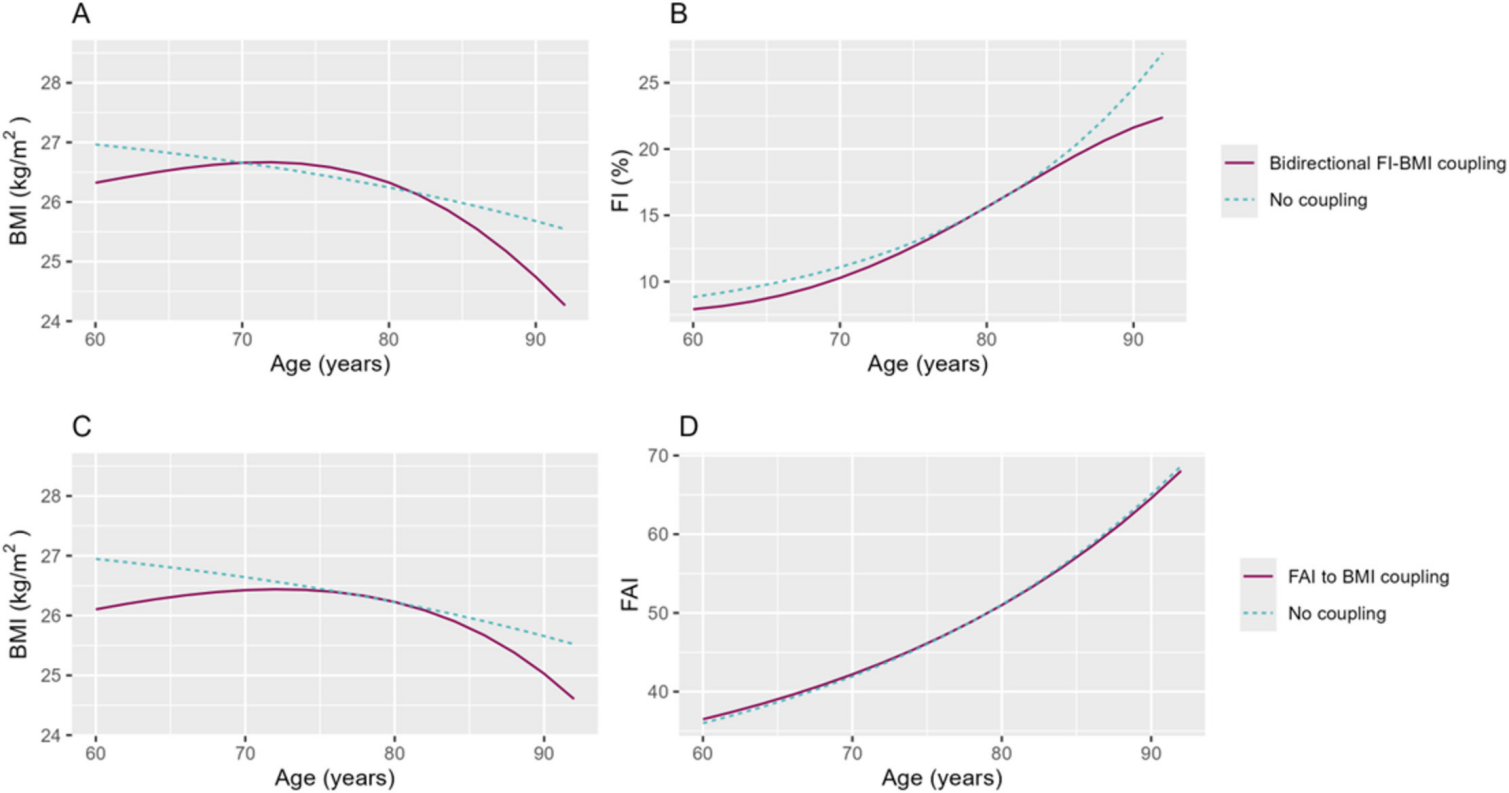
BMI and FI trajectories (A,B) and BMI and FAI trajectories (C,D) from bivariate dual change score models with and without coupling effects. Longitudinal trajectories without coupling effects are shown in blue dotted lines, and trajectories considering coupling are shown in red. Trajectories were generated with estimated mean levels of BMI, FI and FAI at ages 60–91.9 from bivariate dual change score models. All estimates were adjusted for sex, smoking status, study and twin relatedness. BMI, FI and FAI trajectories included a linear slope and a proportional change parameter. The bidirectional FI–BMI coupling model included one coupling parameter linking BMI to FI change and one coupling parameter linking FI to BMI change. The FAI to BMI coupling model was unidirectional and included one coupling parameter linking FAI to BMI change. BMI—body mass index, FAI—functional aging index, FI—frailty index.

In the association between BMI and FI, we selected a full coupling model, which allows for bidirectional associations between BMI and FI (Table S7, *p* values based on LRT ≤ 0.001). The linear slope and proportional change for the BMI trajectory were attenuated in the model with coupling from FI when compared to the no‐coupling model, and the coupling parameter was negative (coupling parameter [*γ*] = −0.04, 95% confidence interval [CI] = −0.05 to −0.03, *p* value ≤ 0.001), indicating that higher FI levels were associated with a more significant BMI decline. Table 2 presents the estimates in coupling and no‐coupling models. Full results, including covariates, variances and covariances, were presented in Table S9. Because FI levels were lower at younger ages and increased exponentially with age, the coupling effects from FI led to a slight increase in BMI at earlier ages, stabilization around age 70–75 and then a steeper decline in BMI at older ages (Figure 2A, which compares the trajectory from the coupling model to that from the no‐coupling model).

**TABLE 2:** Estimates from dual change score models of BMI and FI.

	No coupling		Full coupling	
	Estimate	95% CI	Estimate	95% CI
Mean intercept BMI (*μ*BMI_0_)	26.97*	26.40, 27.53	26.32*	25.76, 26.88
Mean slope BMI (*μ*BMI_slope_)	−1.75*	−2.58, −0.92	−0.36	−1.37, 0.65
Mean intercept FI (*μ*FI_0_)	8.83*	7.37, 10.29	7.91*	6.41, 9.42
Mean slope FI (*μ*FI_slope_)	−0.97*	−1.47, −0.47	−23.44*	−28.89, −17.99
Proportional change parameters (*β*)				
*β*_BMI_	0.06*	0.03, 0.09	0.03	−0.01, 0.07
*β*_FI_	0.15*	0.12, 0.18	0.14*	0.11, 0.17
Coupling parameters (*γ*)				
*γ*_BMI⟶ΔFI_			0.86*	0.65, 1.06
*γ*_FI⟶ΔBMI_			−0.04*	−0.05, −0.03

*Note:* Estimates and 95% confidence intervals were derived from dual change score models with and without bivariate coupling parameters. All models were adjusted for sex, smoking status, study and twin relatedness. The bivariate dual change score model with the best fit was a full coupling model, which included bidirectional coupling with one coupling parameter linking BMI to FI change and one coupling parameter linking FI to BMI change. *μ*BMI_0_ denotes mean BMI intercept, *μ*BMI_slope_ denotes mean BMI slope, *μ*FI_0_ denotes mean FI intercept, *μ*FI_slope_ denotes mean FI linear slope, *β*_BMI_ denotes proportional change of BMI, *β*_FI_ denotes proportional change of FI, *γ*_BMI⟶ΔFI_ denotes coupling parameters from BMI to FI change and *γ*_FI⟶ΔBMI_ denotes coupling parameters from FI to BMI change.

Abbreviations: BMI—body mass index, CI—confidence interval, FI—frailty index.

* *p* < 0.001.

For FI, including coupling from BMI, resulted in a lower intercept level, a stronger negative linear slope, attenuated proportional change and positive coupling effects from BMI (*γ* = 0.86, 95% CI = 0.65–1.06, *p* value ≤ 0.001, Tables 2 and S9). As BMI generally declined with age, this led to a lower initial FI level but a steeper increase in FI until approximately age 75, followed by a more gradual FI increase after age 85 in the coupling model compared to the no‐coupling model (Figure 2B).

We selected a unidirectional model in the association between BMI and FAI, where FAI preceded BMI changes (*p* value based on LRT ≤ 0.001, Table S8). Similar to FI, including coupling from FAI resulted in an attenuated linear slope and proportional change in BMI, along with negative coupling parameters where higher FAI was associated with a greater decline in BMI (*γ* = −0.02, 95% CI = −0.02 to −0.01, *p* value ≤ 0.001, Table 3; full results in Table S10). Like FI, FAI levels were lower at younger ages and increased exponentially with age, accounting for the coupling effects of FAI, therefore led to a slight increase in BMI at earlier ages, followed by a flattening and then a steeper decline in BMI at higher ages (Figure 2C, compares the trajectory from the coupling model to that from the no‐coupling model).

**TABLE 3:** Estimates from dual change score models of BMI and FAI.

	No coupling		FAI to BMI coupling	
	Estimate	95% CI	Estimate	95% CI
Mean intercept BMI (*μ*BMI_0_)	26.95*	26.37, 27.52	26.10*	25.47, 26.74
Mean slope BMI (*μ*BMI_slope_)	−1.77*	−2.67, −0.87	−0.69	−1.57, 0.19
Mean intercept FAI (*μ*FAI_0_)	35.99*	33.85, 38.13	36.49*	34.54, 38.43
Mean slope FAI (*μ*FAI_slope_)	−2.23*	−3.47, −0.99	−2.35*	−3.53, −1.17
Proportional change parameters (*β*)				
*β*_BMI_	0.06*	0.03, 0.10	0.05^	0.02, 0.08
*β*_FAI_	0.09*	0.07, 0.11	0.09*	0.07, 0.11
Coupling parameters (*γ*)				
*γ*_FAI⟶ΔBMI_			−0.02*	−0.02, −0.01

*Note:* Estimates and 95% confidence intervals were derived from dual change score models with and without coupling parameters. All models adjusted for sex, smoking status, study and twin relatedness. The bivariate dual change score model with the best fit was a unidirectional model, with one coupling parameter linking FAI to BMI change. *μ*BMI_0_ denotes mean BMI intercept, *μ*BMI_slope_ denotes mean BMI slope, *μ*FAI_0_ denotes mean FAI intercept, *μ*FAI_slope_ denotes mean FAI linear slope, *β*_BMI_ denotes proportional change of BMI, *β*_FAI_ denotes proportional change of FAI and *γ*_FAI⟶ΔBMI_ denotes coupling parameters from FAI to BMI change.

Abbreviations: BMI—body mass index, CI—confidence intervals, FAI—functional aging index.

^ *p* < 0.01.

* *p* < 0.001.

## 4 Discussion

Longitudinal data and DCSM enabled us to investigate the dynamic relationship between trajectories of BMI and BA at the physiological (FI) and functional (FAI) levels from age 60 to 91.9, providing insights into their temporal changes and direction of associations. We found a bidirectional relationship between BMI and FI such that higher BMI accelerates FI increment, and higher FI drives steeper BMI decline, particularly at higher ages. In contrast, the BMI–FAI relationship was unidirectional, with higher FAI predicting a steeper decline in BMI but no evidence of an effect of BMI on change in FAI.

The relationship between BMI and biological aging is complex and intertwined. In our study, both FI and FAI drove BMI decline, with higher biological aging leading to steeper BMI declines in late life. BMI and biological aging measured as FI exhibited a bidirectional nature, where a higher BMI accelerated biological aging, and accelerated aging, in turn, drove BMI decline.

The finding that higher biological aging drove steeper BMI decline may result from age‐related physiological changes intrinsic to aging, such as decreased lean body mass, bone mass, sense of taste and smell and early satiety [24]. This decline may be intrinsic to the aging process [24]. Alternatively, BMI decline may result from various causes, including intentional weight loss from lifestyle interventions or unintentional elements, the latter being more prevalent among older individuals [24].

Increased biological aging and unintentional weight loss may share common risk factors, including medical, psychosocial and socioeconomic elements [24, 25]. Furthermore, both FI and low BMI, indicative of BMI decline, may have overlapping genetic aetiologies [26]. The common risk factors between BMI decline and accelerated biological aging, whether genetic, medical, psychosocial or socioeconomic, may explain why our findings consistently show that increased biological aging predicted BMI decline. Notably, substantial unintentional weight loss, defined as more than 5% over 6 months to a year, is associated with underlying comorbidities and increased mortality risk [24, 27]. Therefore, a sharp BMI decline likely signals the onset of health issues or disease progression, highlighting the need for closer clinical attention.

Higher BMI, in turn, accelerated the rate of FI increment, aligning with previous studies that have shown an association between obesity and increased risk of frailty [28, 29] and a causal role of obesity in the development of frailty [30] as well as other measures of accelerated aging [31]. FI can serve as a proxy for aging at the whole organism level [8], and together with previous research, our findings support the hypothesis that obesity accelerates biological aging. Obesity is a risk factor for age‐related diseases such as hypertension, type II diabetes and cardiovascular diseases, all of which are conditions captured by FI. Therefore, high BMI may accelerate aging, as measured by FI, through predisposition to age‐related conditions.

Our findings align with research suggesting that the obesity paradox may result from reverse causality [15]. We found that BMI declined more steeply at older ages due to increasing FI and FAI, reflecting health deterioration. These trajectories can create the illusion that higher BMI is protective, while in actuality, lower BMI signals poorer health, explaining the link between low BMI and worse outcomes.

Simultaneously, we found that higher BMI accelerated biological aging, as measured by FI, underscoring the adverse long‐term effects of obesity. This effect was mainly seen in younger ages but weakened at higher ages as BMI declined. These findings challenge the interpretations of the obesity paradox that suggest high BMI is protective in aging populations. Instead, they align with previous research indicating that the apparent link between obesity and better health outcomes in older age is likely explained by reverse causality—where advanced aging or ill health drives weight loss and inflates the association between high BMI and better health—rather than a genuine protective effect of obesity [15, 27]. Future research should focus on the health implications of BMI and weight changes instead of cross‐sectional measures to better understand the obesity paradox.

In contrast with the BMI–FI association, the BMI–FAI association was unidirectional, with no evidence of a coupling effect of BMI on change in FAI. FI and FAI are correlated but complementary measures of biological aging [17]. While FI captures changes in physiological function from multiple body systems and aspects of functional capacity, FAI focuses on measuring changes in functional capacity that impact mobility and independence [17]. FI trajectories increased earlier and more sharply than FAI. Because we only estimated the coupling parameters for associations between BMI level and change in FI or FAI over 2 years, it is possible that more long‐term effects were not captured.

Ferrucci et al. have proposed a hierarchical view of aging metrics that begins with molecular and cellular aging, followed by phenotypic and functional aging [32]. Because FAI pertains specifically to the functional aging category, whereas FI includes deficits related to not only functional measures (e.g., activities to daily living) but also age‐related signs and symptoms, reflecting both functional and phenotypic aging stages, the effects of BMI on biological aging may manifest earlier in FI than in FAI. Consequently, the functional aspects of aging, as measured by FAI, may be observed later. These differences in FI and FAI and the hierarchal view of aging metrics may explain the differing direction in their associations with BMI. Additionally, pathophysiological changes common to obesity and aging, such as increased systemic inflammation, mitochondrial dysfunction and cellular senescence [5, 6], are more closely related to the molecular and cellular aging metrics. Therefore, it is plausible that a higher BMI only influences the earlier stages of aging, with no direct effects on the later functional stage.

While our findings align with the growing evidence that high BMI accelerates biological aging, the biological mechanisms underlying this association remain unclear; that said, it likely involved multiple, interconnected pathways [5, 6, 33]. A recent multicohort study has linked obesity to several biological hallmarks of aging, such as deregulated nutrient sensing, telomere attrition, epigenetic alterations, mitochondrial dysfunction, impaired intercellular communication (inflammaging) and cellular senescence and the co‐occurrence of age‐related diseases highlighting the role of obesity in advancing cellular aging and the onset of age‐related diseases [33]. Based on experimental and observational evidence, Tam et al. highlighted obesity's role in redox imbalance and mitochondrial dysfunction, which may drive oxidative stress, systemic inflammation and cellular damage, accelerating biological aging [5]. These obesity‐associated disturbances may be driven by excess glucose and free fatty acids, which promote electron leakage from mitochondria, NOX activation and weakened antioxidant defences through glutathione depletion [34]. Diaz‐Ruiz et al. further underscored the pathophysiological parallels between visceral obesity and aging, particularly how adiposity may promote insulin resistance, metabolic dysfunction and chronic inflammation through the secretion of cytokines and chemokines such as TNF‐*α* and IL‐6, which drive systemic inflammation [6]. Taken together, these mechanisms suggest that high BMI is not merely a risk factor for age‐related diseases but may actively accelerate biological aging. Future research should explore these pathways to identify interventions to mitigate obesity and aging.

The current study leveraged rich longitudinal data to capture the dynamic interplay of fluctuating health factors across an age range from 60.0 to 91.9 years, made possible by the powerful DCSM. The study benefited from objective measures, including weight, height, peak respiratory flow, grip strength and gait speed, all assessed by trained healthcare professionals following a standardized protocol. Additionally, information on subjective health measures was gathered through self‐reported questionnaires.

However, our study was limited by the lack of longitudinal data spanning ages 60.0–91.9 years for the whole sample. Baseline ages in GENDER and OCTO‐Twin were higher than those in the SATSA. Hence, the twins in the GENDER and OCTO‐Twin studies were likely to be survivors who underwent healthier aging trajectories. This may introduce survival bias, as twins with increased biological aging, greater frailty or multiple comorbidities may be excluded from this analysis due to premature mortality. Consequently, the observed associations between BMI and biological aging may primarily reflect healthier, older twins rather than those more vulnerable and may be lost to follow‐up due to early mortality. However, we attempted to limit the survival bias by adjusting for study.

Additionally, we assumed no variation in proportional change across ages in our models, as breakpoints that allow proportional change and coupling effects to vary caused model instability. However, the DCSM, with or without breakpoints, is an exponential model that captures non‐linearity and dynamic associations through proportional effects and coupling parameters.

Furthermore, due to the limited sample size, we limited the age range to 60.0–91.9 and did not examine how the associations and trajectories may differ by sex or other risk factors. BMI and biological aging trajectories may vary by sex, potentially influencing their bivariate associations. However, previous research examining sex differences in BMI and FI trajectories using Swedish Twin cohorts with larger sample sizes found no differences in BMI and FI trajectories between males and females, except that males tended to have higher BMI [35]. In contrast, females tended to have higher FI [11].

Another limitation is using BMI as a proxy for adiposity, as it does not measure fat distribution or differentiate fat from lean mass [10]. Measures such as waist circumference are more direct assessments of visceral fat level, which is strongly linked to cardiovascular health [36]. Still, BMI is a widely available and effective measure of obesity at the population level and has been shown to capture critical late‐life changes, like unintentional weight loss, as effectively as the other alternative methods [37]. BMI detects declines in overall body mass, making it an appropriate indicator of health changes in late life. Nevertheless, BMI alone may not provide a comprehensive understanding of how adiposity influences late‐life health [38]. Future studies should consider multiple measures of adiposity, including waist circumference and body composition assessments, to provide a more nuanced understanding of the impact of body composition and adiposity on health among older people.

## 5 Conclusions

Our study highlights a complex and dynamic relationship between BMI and biological aging in late life. At the physiological level, BMI and aging exhibit bidirectional associations: Higher BMI accelerates physiological aging, and higher physiological aging, in turn, accelerates BMI decline. At the functional level, we observed a unidirectional relationship: Higher functional aging accelerates BMI decline, but higher BMI does not accelerate functional aging. Aging at both levels accelerates BMI decline, indicating unintentional weight loss may signal accelerated aging. A higher BMI accelerates aging on the physiological level, substantiating the hypothesis that obesity accelerates the aging process. However, no such effect was seen for functional aging, indicating that the relationship between BMI and aging may be present only at certain levels of aging. These findings underscore the intricate relationship between BMI and aging and the importance of maintaining a healthy BMI by managing both obesity and unintentional weight loss and preserving functional capacity for healthy aging.

## Ethics Statement

All participants provided written informed consent. The Research Ethics Committee of Stockholm approved this study (2015/1729–35/5, 2022‐06634‐01, 2024‐03706‐0). All authors of this manuscript certify that they comply with the ethical guidelines for authorship and publishing in the Journal of Cachexia, Sarcopenia, and Muscle [39].

## Conflicts of Interest

The authors declare no conflicts of interest.

## Supporting information


**Figure S1.** Data collection timeline.
**Table S1.** Items included in the computation of frailty index by study.
**Figure S2.** Statistical analytical workflow.
**Table S2.** Number of participants by total number of observations.
**Table S3.** Model comparisons of univariate dual change score models.
**Table S4.** Estimates and 95% confidence interval from univariate dual change score model of BMI in frailty index and functional aging index analytic sample.
**Figure S3.** BMI age trajectory in FI analytic sample.
**Figure S4.** BMI age trajectory in FAI analytic sample.
**Table S5.** Estimates and 95% confidence interval of parameters from univariate dual change score model of frailty index.
**Table S6.** Estimates and 95% confidence interval of parameters from univariate dual change score model of functional aging index.
**Figure S5.** Age trajectory of frailty index.
**Figure S6.** Age trajectory of functional aging index.
**Table S7.** Model comparisons of bivariate dual change score models of BMI and frailty index.
**Table S8.** Model comparisons of bivariate dual change score models of BMI and functional aging index.
**Table S9.** Estimates from dual change score models of BMI and frailty index, including variances and covariances.
**Table S10.** Estimates from dual change score models of BMI and functional aging index including variances and covariances.

## Data Availability

Data are not publicly available due to ethical restrictions but can be applied through NEAR: https://near‐aging.se/.
