# Supplementary material for: A Longitudinal Study of the Bidirectional Temporal Dynamics Between Body Mass Index and Biological Aging
Source: J Cachexia Sarcopenia Muscle. 2025 May 8;16(3):e13824. doi: 10.1002/jcsm.13824 (PMC12059470; doi:10.1002/jcsm.13824)
Supplement: Supplementary file 1 — Figure S1. Data collection timeline. Table S1. Items included in the computation of frailty index by study. Figure S2. Statistical analytical workflow. Table S2. Number of participants by total number of observations. Table S3. Model comparisons of univariate dual change score models. Table S4. Estimates and 95% confidence interval from univariate dual change score model of BMI in frailty index and functional aging index analytic sample. Figure S3. BMI age trajectory in FI analytic sample. Figure S4. BMI age trajectory in FAI analytic sample. Table S5. Estimates and 95% confidence interval of parameters from univariate dual change score model of frailty index. Table S6. Estimates and 95% confidence interval of parameters from univariate dual change score model of functional aging index. Figure S5. Age trajectory of frailty index. Figure S6. Age trajectory of functional aging index. Table S7. Model comparisons of bivariate dual change score models of BMI and frailty index. Table S8. Model comparisons of bivariate dual change score models of BMI and functional aging index. Table S9. Estimates from dual change score models of BMI and frailty index, including variances and covariances. Table S10. Estimates from dual change score models of BMI and functional aging index including variances and covariances. [file JCSM-16-e13824-s001.pdf]

Supplemental materials

A Longitudinal Study of the Bidirectional Temporal Dynamics between Body Mass Index and Biological Aging

Authors:
*Peggy Ler, Ph.D.[1], Jonathan K. L. Mak, Ph.D.[1,2], Chandra A. Reynolds, Ph.D. [3], Alexander Ploner, Ph.D.[1], Nancy L. Pedersen, Ph.D. [1], Juulia Jylhävä, Ph.D.[1,4,5], Anna K. Dahl Aslan, Ph.D.[6], Deborah Finkel, Ph.D.[7,8], Ida K. Karlsson, Ph.D.[1]
Affiliations:
[1] Department of Medical Epidemiology and Biostatistics, Karolinska Institutet, Nobels väg 12A, 17165 Solna, Sweden
[2] Department of Pharmacology and Pharmacy, Li Ka Shing Faculty of Medicine, The University of Hong Kong, Hong Kong SAR
[3] Institute for Behavioral Genetics and Department of Psychology and Neuroscience, University of Colorado Boulder, Boulder, Colorado, USA
[4] Faculty of Medicine and Health Technology and Gerontology Research Center, University of Tampere, 33520 Tampere, Finland
[5] Tampere Institute for Advanced Study, Tampere, Finland
[6] School of Health Sciences, University of Skövde, 54128 Skövde, Sweden
[7] Center for Economic and Social Research, University of Southern California, 635 Downey Way, Los Angeles, California 90089-3332, USA
[8] Institute for Gerontology, Jönköping University, Jönköping, 55111Sweden

Table of contents

Figure S1: Data collection timeline Pg 2

Table S1: Items included in the computation of frailty index by study Pg 3

Figure S2: Statistical analytical workflow Pg 5

Table S2: Number of participants by total number of observations Pg 6

Table S3: Model comparisons of univariate dual change score models Pg 7

Table S4: Estimates and 95% confidence interval from univariate dual change score model of BMI in frailty index and functional aging index analytic sample Pg 8

Figure S3: BMI age trajectory in FI analytic sample Pg 9

Figure S4: BMI age trajectory in FAI analytic sample Pg 10

Table S5: Estimates and 95% confidence interval of parameters from univariate dual change score model of frailty index Pg 11

Table S6: Estimates and 95% confidence interval of parameters from univariate dual change score model of functional aging index Pg 12

Figure S5: Age trajectory of frailty index Pg 13

Figure S6: Age trajectory of functional aging index Pg 14

Table S7: Model comparisons of bivariate dual change score models of BMI and frailty index Pg 15

Table S8: Model comparisons of bivariate dual change score models of BMI and functional aging index Pg 16

Table S9: Estimates from dual change score models of BMI and frailty index, including variances and covariances Pg 17

Table S10: Estimates from dual change score models of BMI and functional aging index including variances and covariances Pg 20

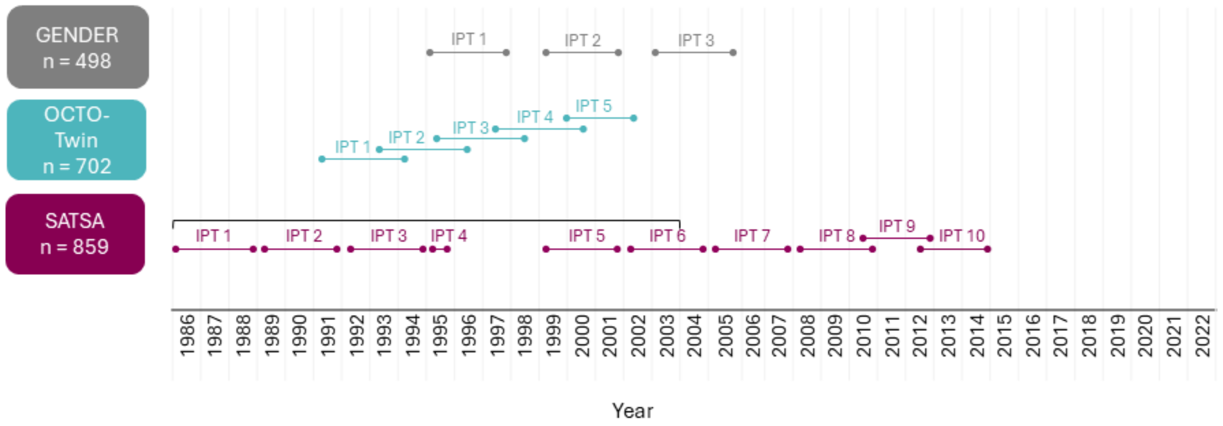

Figure S1: Data Collection Timeline

Abbreviation: IPT - in-person testing

Table S1: Items included in the computation of frailty index by study

Items	SATSA	OCTO-Twin	GENDER
General health status	✓	✓	✓
Limited from doing things one would normally like to do due to health status	✓	✓	✓
Cancer or leukemia	✓		✓
Rheumatoid arthritis	✓	✓	✓
Arthritis	✓	✓	
Chronic bronchitis or emphysema	✓	✓	
Cataracts	✓	✓	✓
Chest pain	✓	✓	✓
Circulation problems in arms or legs	✓	✓	✓
Persistent cough	✓		
Goiter or other gland problems	✓	✓	✓
Heart failure	✓	✓	✓
Hypertension	✓	✓	✓
Kidney disease	✓	✓	✓
Osteoporosis	✓	✓	✓
Sciatica	✓	✓	✓
Anemia	✓	✓	
Cerebral hemorrhage or blood clot in brain	✓	✓	
Dizziness	✓	✓	
Gastric ulcer	✓	✓	✓
Allergies/allergic manifestations	✓	✓	
Asthma	✓	✓	
Showering and bathing	✓	✓	✓
Getting in and out of bed	✓		✓
Dressing and undressing	✓		✓
Self-grooming	✓		
Walking	✓	✓	✓
Trouble getting to the toilet in time	✓	✓	✓
Traveling further distances	✓		✓
Housework	✓		✓
Preparing meals	✓		

**Table S1 continue**			
**Items**	**SATSA**	**OCTO-Twin**	**GENDER**
Managing medications	✓		✓
Managing money	✓		
Using telephone	✓		✓
Grocery shopping	✓		✓
Hearing acuity	✓	✓	
Vision acuity	✓	✓	
Feeling lonely the past week	✓	✓	✓
Feeling depressed the past week	✓		✓
Feeling happy the past week	✓		
Feeling tired the past week	✓		
Keeping body fit		✓	
Heart attack		✓	✓
Vascular spasm in leg		✓	✓
Herpes		✓	
Migraine		✓	✓
Glaucoma		✓	✓
Speech impairment		✓	✓
Eczema		✓	✓
Hip joint impairment		✓	✓
Neck pain		✓	
Shoulder pain		✓	
Gall bladder		✓	✓
Insomnia		✓	✓
Psychological problems		✓	
Stroke			✓
Epilepsy			✓
Liver disease			✓
Gout			✓
Picking something up from the floor			✓
Handling small things with your fingers			✓
Type II Diabetes	✓	✓	✓
Total number of items	42	41	42

An '✓' in the table's cell represents the item's presence in the calculation of frailty index.

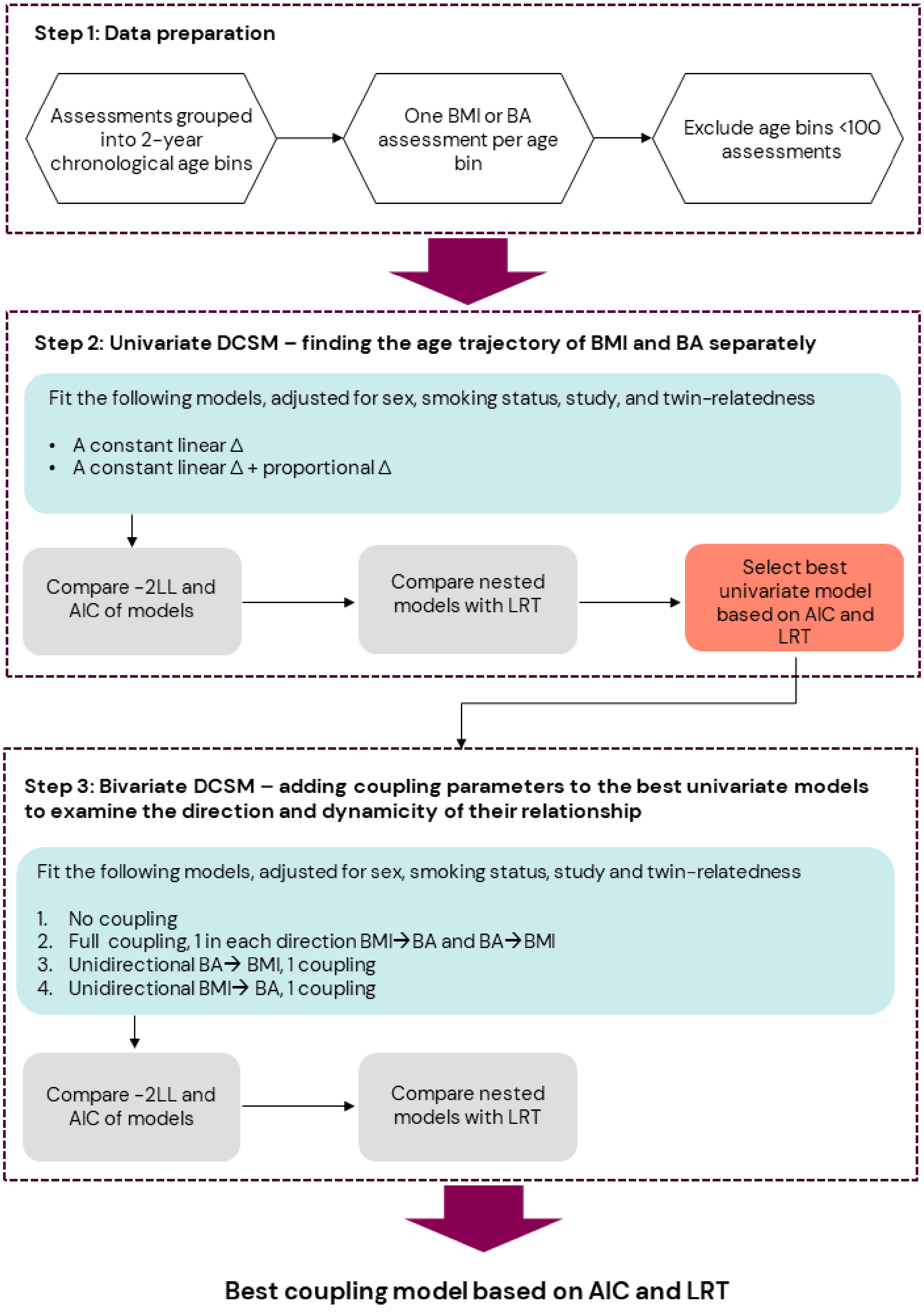

Figure S2: Statistical analytical workflow

Figure illustrates the statistical analytical workflow adopted in the study. Δ denotes change.

Abbreviations: AIC- Aikake information criteria, BA – biological age, BMI – body mass index, LRT – likelihood ratio tests of nested models

Table S2: Number of participants by total number of observations

N. of observations	FI sample	FAI sample
1	382	376
2	303	319
3	597	547
4	207	198
5	241	219
6	126	126
7	47	48
8	44	41
9	27	26
10	2	2
Total N. of observations	6512	6216
N. of individuals with >= 1 observation	1976	1902
N. of individuals with >= 3 observations	1291	1207

The number of observations during follow-up from age 60.0 to 92.0 in the entire FI and FAI analytical sample.

Abbreviations: FAI – functional aging index, FI – frailty index, N. – number

Table S3: Model comparisons of univariate dual change score models of BMI in FI and FAI analytical samples, FI and FAI

Variable	Models	N. of parameters	-2LL	AIC	LRT p-value
BMI in the FI analytical sample	Proportional effects	22	39349.65	39393.65	Base models
	No proportional effects	21	39367.25	39409.25	<0.001
BMI in the FAI analytical sample	Proportional effects	22	38917.27	38961.27	Base models
	No proportional effects	21	38934.17	38976.17	<0.001
FI	Proportional effects	22	50787.84	50831.84	Base models
	No proportional effects	21	50890.9	50932.9	<0.001
FAI	Proportional effects	22	45415.84	45459.84	Base models
	No proportional effects	21	45483.68	45525.68	<0.001

Univariate dual change score models tested were adjusted for sex, smoking status, study, and twin-relatedness. The model with no breakpoint was compared to a model with no proportional change effects. A p-value below 0.05 in the likelihood ratio test of nested models indicates that the compared models are significantly different and that the base model is a better fit for the data.

Abbreviations: AIC – Akaike's information criteria, N. – number, -2LL – log-likelihood ratio, LRT – likelihood ratio test of nested models

Table S4: Estimates and 95% confidence interval from univariate dual change score model of BMI in frailty index and functional aging index analytic sample

	FI analytic sample		FAI analytic sample	
	**Estimate**	**95% CI**	**Estimate**	**95% CI**
Mean intercept BMI (μBMI_0)	26.91	26.36,27.47	26.92	26.35,27.49
Mean slope BMI (μBMI_{slope})	-1.81	-2.64,-0.98	-1.78	-2.60,-0.95
Proportional change parameters (β)				
β_{BMI}	0.07	0.03,0.10	0.06	0.03,0.10
Variances and covariances (σ^2)				
Variance intercept	9.76	8.2,11.33	9.75	8.18,11.32
Covariance intercept-slope	-0.83	-1.14,-0.52	-0.81	-1.12,-0.5
Variance slope	0.09	0.05,0.13	0.09	0.05,0.13
Residual variance	1.54	1.46,1.62	1.54	1.46,1.62
Parameters of covariates				
Mean sex	0.6	0.57,0.62	0.59	0.57,0.62
Variance sex	0.24	0.23,0.26	0.24	0.23,0.26
Sex on intercept	-0.26	-0.77,0.26	-0.26	-0.79,0.27
Sex on slope	-0.002	-0.07,0.06	-0.003	-0.07,0.06
Mean smoking	0.48	0.46,0.5	0.49	0.46,0.51
Smoking variance	0.25	0.23,0.27	0.25	0.23,0.27
Smoking on intercept	-0.63	-1.12,-0.13	-0.63	-1.13,-0.13
Smoking on slope	0.03	-0.03,0.09	0.03	-0.03,0.09
Mean study	-0.18	-0.23,-0.13	-0.22	-0.28,-0.17
Study variance	1.31	1.23,1.39	1.31	1.23,1.39
Study on intercept	0.12	-0.14,0.38	0.12	-0.13,0.38
Study on slope	-0.05	-0.08,-0.01	-0.05	-0.08,-0.02
Parameters for twin pairs (σ^2)				
Variance intercept	6.69	5.05,8.33	6.71	5.06,8.36
Covariance intercept-slope	-0.54	-0.78,-0.29	-0.53	-0.77,-0.29
Variance slope	0.05	0.02,0.08	0.05	0.02,0.08

Estimates and 95% confidence intervals derived from univariate dual change score models adjusted for sex, smoking, study, and twin-relatedness, with age as the underlying time scale. Each models included one proportional change parameter (β), where β_{BMI} denotes the proportional change parameter of body mass index. μBMI_0 denotes the mean intercept of body mass index, μBMI_{slope} denotes the mean BMI linear slope.

Abbreviations: BMI – body mass index, CI – confidence interval, FAI – functional aging index, FI – frailty index

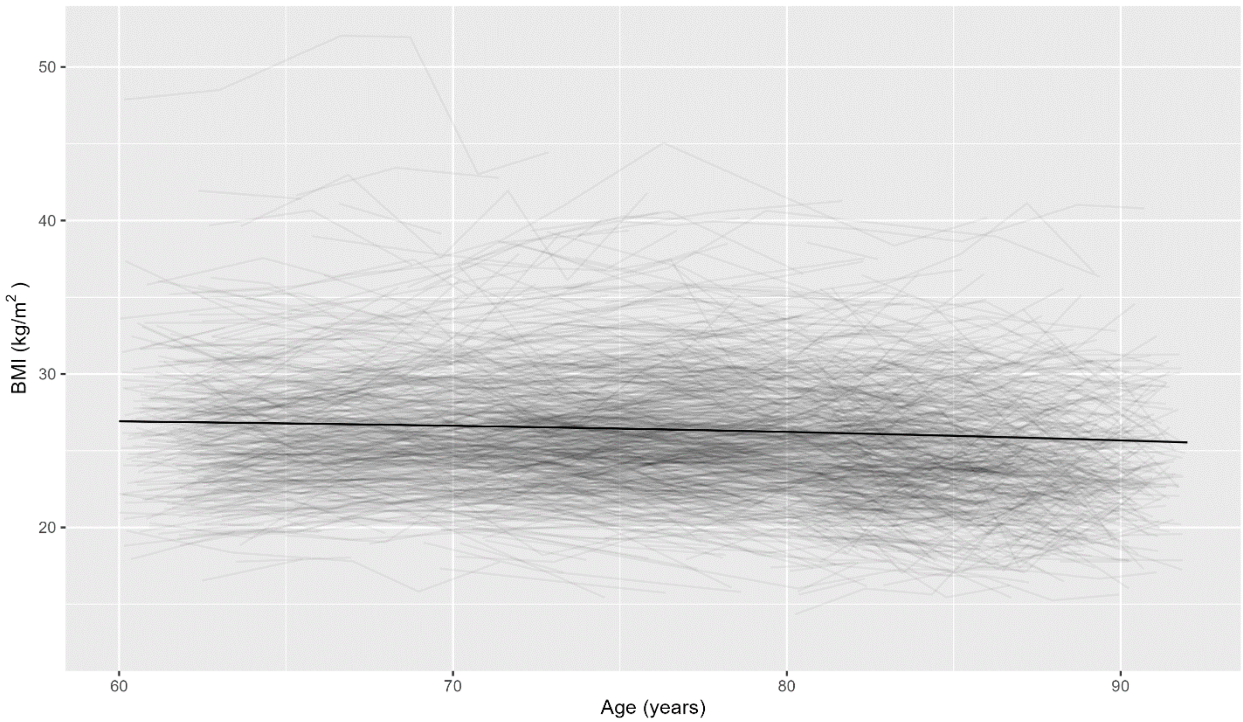

Figure S3: BMI age trajectory in FI analytical sample

Black line denotes the BMI trajectory from univariate dual change score models adjusted for sex, smoking, study, and twin-relatedness, with age as the underlying time scale. The model included one proportional change parameters (β). Grey lines represent the BMI trajectory of individuals within the FI analytical sample.

Abbreviations: BMI – body mass index, FI – frailty index

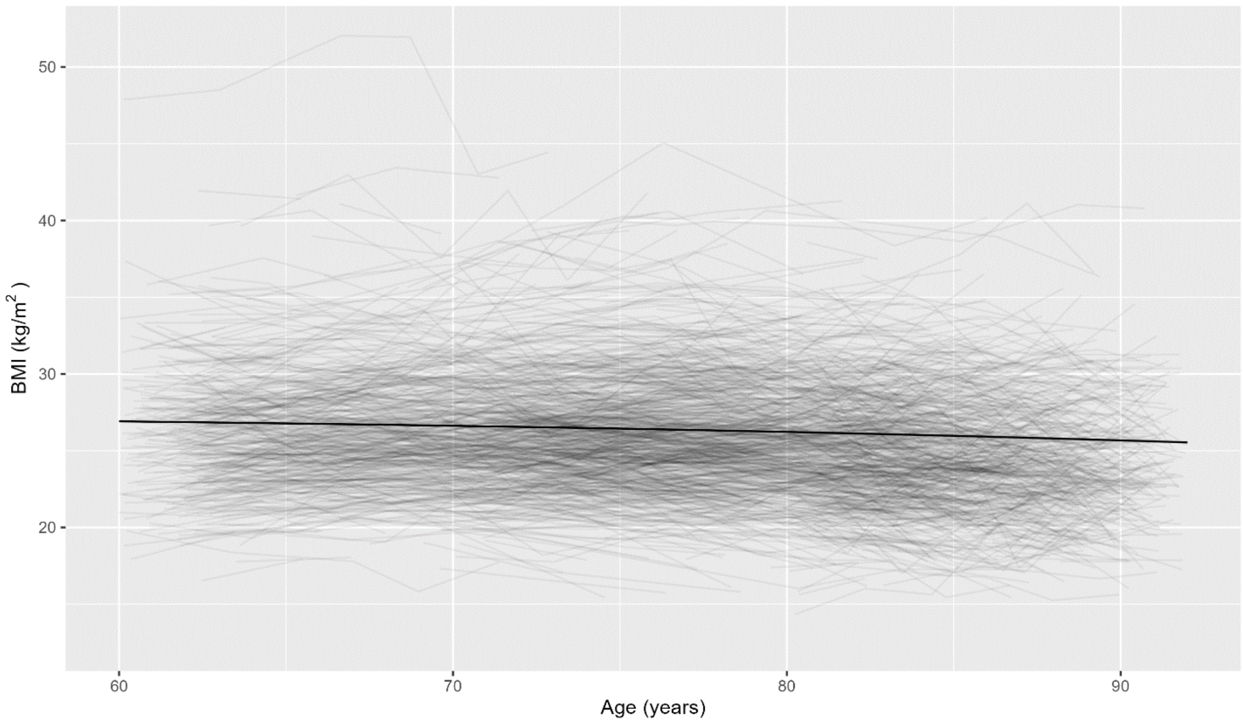

Figure S4: BMI age trajectory in FAI analytical sample

Black line denotes the BMI trajectory from univariate dual change score models adjusted for sex, smoking, study, and twin-relatedness, with age as the underlying time scale. Models included one proportional change parameter (β). Grey lines represent the BMI trajectory of individuals within the FAI analytical sample.

Abbreviations: BMI – body mass index, FAI – functional aging index

Table S5: Estimates and 95% confidence interval of parameters from univariate dual change score model of frailty index

	Estimate	95% CI
Mean intercept FI (μFI_0)	9.09	7.65,10.53
Mean slope FI (μFI_{slope})	-1.10	-1.6,-0.60
Proportional change parameters (β)		
β_{FI}	0.16	0.13,0.18
Variances and covariances (σ^2)		
Variance intercept	36.02	26.33,45.71
Covariance intercept-slope	-5.95	-8.07,-3.82
Variance slope	1.04	0.58,1.51
Residual variance	21.03	19.82,22.24
Parameters of covariates		
Mean sex	0.60	0.57,0.62
Variance sex	0.24	0.23,0.26
Sex on intercept	3.17	1.95,4.40
Sex on slope	-0.53	-0.79,-0.28
Smoking on intercept	0.48	0.46,0.50
Smoking on slope	0.25	0.23,0.27
Mean smoking	0.70	-0.54,1.94
Smoking variance	-0.07	-0.31,0.16
Mean study	-0.18	-0.23,-0.13
Study variance	1.31	1.23,1.39
Study on intercept	2.44	1.87,3.01
Study on slope	-0.43	-0.58,-0.29
Parameters for twin pairs (σ^2)		
Variance intercept	23.17	13.36,32.99
Covariance of intercept-slope	-4.08	-6.04,-2.13
Variance slope	0.74	0.33,1.15

Estimates and 95% confidence intervals from univariate dual change score models adjusted for sex, smoking, study and twin-relatedness, with age as the underlying time scale. Model included one proportional change parameter (β), whereby β_{FI} denotes the proportional change parameter of frailty index. μFI_0 denotes the mean intercept of frailty index (FI), μFI_{slope} denotes the mean FI linear slope.

Abbreviations: FI – frailty index, CI – confidence interval

Table S6: Estimates and 95% confidence interval of parameters from univariate dual change score model of functional aging index

	Estimate	95% CI
Mean FAI_0 (μFAI_0)	36.16	34.21,38.11
Mean FAI_{slope} (μFAI_{slope})	-2.42	-3.60,-1.23
Proportional parameters (β)		
β_{FAI}	0.09	0.07,0.12
Variances and covariances (σ^2)		
Variance intercept	42.3	28.39,56.21
Covariance intercept – slope	-4.47	-6.72,-2.23
Variance slope	0.64	0.28,0.99
Residual variance	33.34	31.43,35.25
Parameters of covariates		
Mean sex	0.59	0.57,0.62
Variance sex	0.24	0.23,0.26
Sex on intercept	4.53	3.08,5.98
Sex on slope	-0.49	-0.74,-0.25
Mean smoking	0.49	0.46,0.51
Variance smoking	0.25	0.23,0.27
Smoking on intercept	0.69	-0.74,2.11
Smoking on slope	-0.01	-0.23,0.20
Mean study	-0.22	-0.28,-0.17
Study variance	1.31	1.23,1.39
Study on intercept	-3.00	-3.8,-2.21
Study on slope	0.27	0.17,0.37
Parameters for twin pairs (σ^2)		
Variance intercept	16.01	2.53,29.49
Covariance of intercept-slope	-1.40	-3.52,0.72
Variance slope	0.12	-0.21,0.45

Estimates and 95% confidence intervals from univariate dual change score models adjusted for sex, smoking, study, and twin-relatedness, with age as the underlying time scale. Model included one proportional change parameter (β), whereby β_{FAI} denotes the proportional change parameter of functional aging index (FAI). μFAI_0 denotes the mean intercept of FAI, μFAI_{slope} denotes the mean FAI linear slope.

Abbreviations: FAI – functional aging index, CI – confidence interval

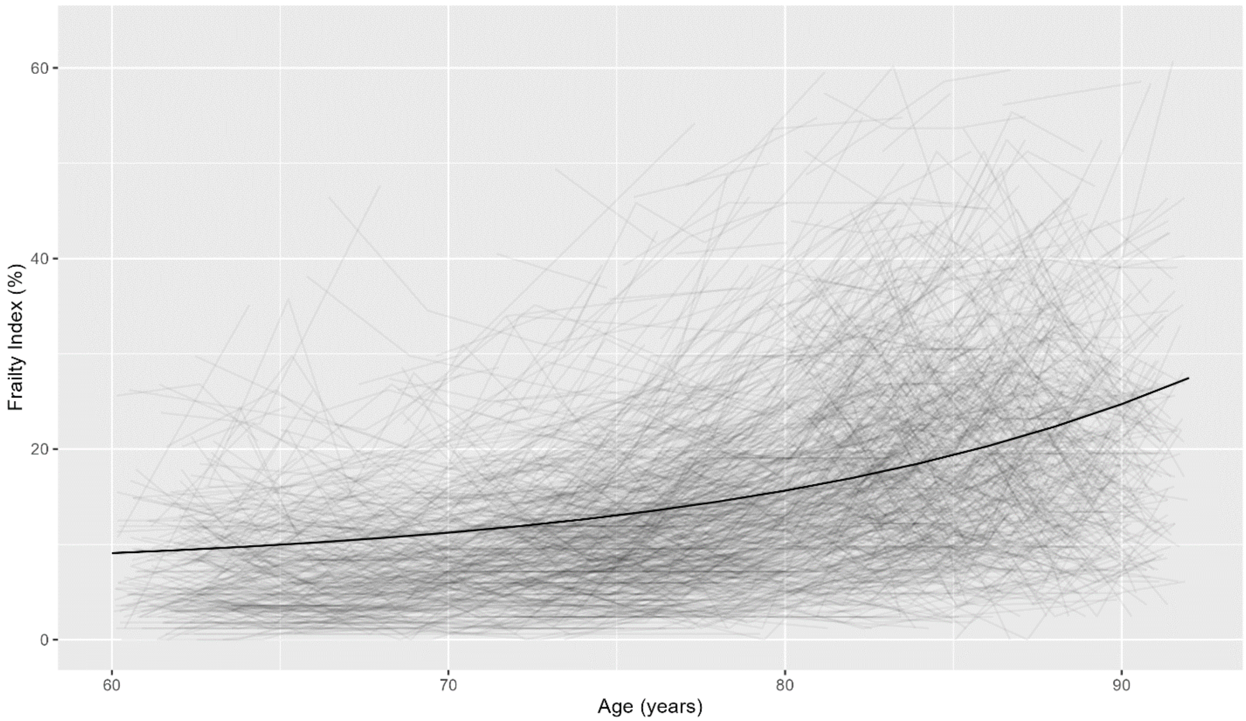

Figure S5: Age trajectory of frailty index

Black line denotes the frailty index (FI) trajectory from univariate dual change score models adjusted for sex, smoking, study, and twin-relatedness, with age as the underlying time scale. Model included one proportional change parameter (β). Grey lines represent each individuals' FI trajectory

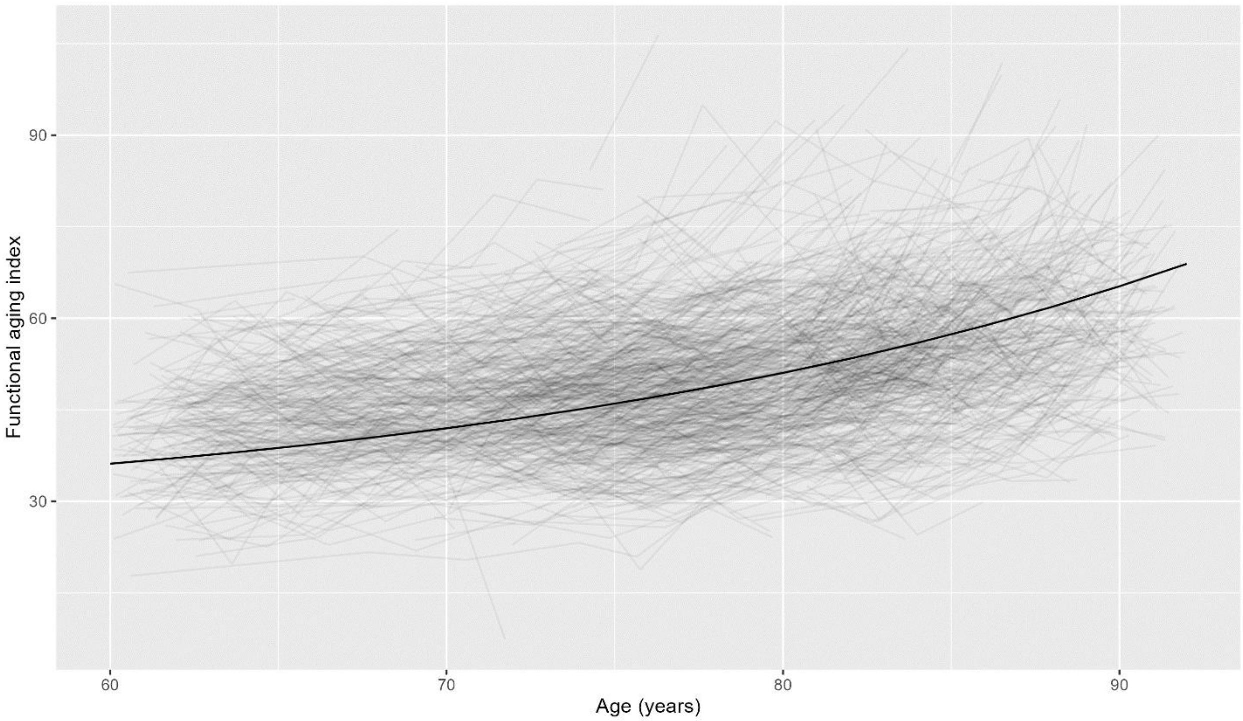

Figure S6: Age trajectory of functional aging index

Black line denotes the functional aging index (FAI) trajectory from univariate dual change score models adjusted for sex, smoking, study, and twin-relatedness, with age as the underlying time scale. Model included one proportional change parameter (β). Grey lines represent each individuals' FAI trajectory

Table S7: Model comparisons of bivariate dual change score models of BMI and frailty index

Models	N. of parameters	-2LL	AIC	LRT p-value
Full coupling	49	77875.46	77973.46	Base model
BMI to FI coupling	48	77957.66	78053.66	<0.001
FI to BMI coupling	48	77964.45	78060.45	<0.001
No coupling	47	78022.06	78116.06	<0.001

All models were adjusted for sex, smoking status, study, and twin-relatedness. 'Full' model indicates coupling in both directions. A p-value from the likelihood ratio test for nested models greater than 0.05 indicated that the compared models were not significantly different, and thus, the simpler model was preferred. The table demonstrates that a full coupling model is significantly different from unidirectional and no coupling models, suggesting that the best-fit model requires coupling in both directions.

Abbreviations: AIC – Akaike's information criteria, BMI – body mass index, FI – frailty index, N. – number, -2LL – log-likelihood ratio, LRT – likelihood ratio test of nested models

Table S8: Model comparisons of bivariate dual change score models of BMI and functional aging index

Models	N. of parameters	-2LL	AIC	LRT p-value
Full coupling	49	72632.12	72730.12	Base model
BMI to FAI coupling	48	72670.79	72766.79	<0.001
FAI to BMI coupling	48	72633.12	72729.12	0.32
No coupling	47	72670.89	72764.89	<0.001
FAI to BMI coupling	48	72633.12	72729.12	Base model
No coupling	47	72670.89	72764.89	<0.001

All models were adjusted for sex, smoking status, study, and twin-relatedness. 'Full' model indicates coupling in both directions. A p-value from the likelihood ratio test of nested models greater than 0.05 indicates that the compared models were not significantly different, favoring the simpler model. The table demonstrates that a full coupling model is not significantly different from a unidirectional model with coupling from FAI to BMI change; therefore, the latter model was preferred for best fit.

Abbreviations: BMI – body mass index, FAI – functional aging index, N. – number, -2LL – log-likelihood ratio, LRT – likelihood ratio tests

Table S9: Estimates from dual change score models of BMI and frailty index, including variances and covariances

	No coupling		Full coupling	
	Estimate	95% CI	Estimate	95% CI
Mean BMI intercept (μBMI_0)	26.97	26.4,27.53	26.32	25.76,26.88
Mean BMI slope (μBMI_{slope})	-1.75	-2.58,-0.92	-0.36	-1.37,0.65
Mean FI intercept (μFI_0)	8.83	7.37,10.29	7.91	6.41,9.42
Mean FI slope (μFI_{slope})	-0.97	-1.47,-0.47	-23.44	-28.89,-17.99
Proportional change parameters (β)				
β_{BMI}	0.06	0.03,0.09	0.03	-0.01,0.07
β_{FI}	0.15	0.12,0.18	0.14	0.11,0.17
Coupling paramerters (γ)				
$\gamma_{BMI \rightarrow \Delta FI}$			0.86	0.65,1.06
$\gamma_{FI \rightarrow \Delta BMI}$			-0.04	-0.05,-0.03
Variances and covariances (σ^2)				
Variance BMI_0	9.77	8.18,11.37	9.25	7.84,10.65
Covariance BMI_0 & BMI_{slope}	-0.81	-1.13,-0.50	-0.38	-0.75,-0.02
Variance BMI_{slope}	0.09	0.05,0.13	0.08	0.04,0.11
Variance residual BMI	1.54	1.47,1.62	1.60	1.51,1.69
Variance FI_0	35.64	25.93,45.35	31.36	23.14,39.59
Covariance FI_{slope} & FI_0	-5.66	-7.72,-3.6	-4.61	-7.27,-1.96
Variance FI_{slope}	0.97	0.53,1.40	6.88	3.70,10.06
Variance residual FI	21.22	19.99,22.45	19.97	18.81,21.12
Covariance residuals BMI & FI	-0.53	-0.77,-0.29	-0.50	-0.75,-0.24
Covariance BMI_0 & FI_0	0.74	-1.44,2.91	-0.20	-2.77,2.36
Covariance BMI_0 & FI_{slope}	-0.08	-0.5,0.34	-7.37	-9.34,-5.39
Covariance FI_0 & BMI_{slope}	-0.10	-0.38,0.18	1.23	0.79,1.67

Covariance FI_{slope} & BMI_{slope}	0.01	-0.04,0.06	0.08	-0.24,0.41
Parameters of covariates				
Mean sex	0.60	0.57,0.62	0.60	0.57,0.62
Variance sex	0.24	0.23,0.26	0.24	0.23,0.26
Sex on BMI_0	-0.27	-0.80,0.25	-0.31	-0.79,0.17
Sex on BMI_{slope}	-0.001	-0.06,0.06	0.09	0.03,0.15
Sex on FI_0	3.27	2.06,4.47	2.44	1.29,3.58
Sex on FI_{slope}	-0.53	-0.77,-0.29	-0.01	-0.42,0.39
Mean smoking	0.48	0.46,0.50	0.48	0.46,0.50
Variance smoking	0.25	0.23,0.27	0.25	0.23,0.27
Smoking on BMI_0	-0.66	-1.16,-0.16	-0.75	-1.23,-0.28
Smoking on BMI_{slope}	0.03	-0.03,0.09	0.04	-0.01,0.10
Smoking on FI_0	0.76	-0.52,2.04	0.26	-0.85,1.36
Smoking on FI_{slope}	-0.08	-0.32,0.16	0.68	0.27,1.09
Mean study	-0.18	-0.23,-0.13	-0.18	-0.23,-0.13
Variance study	1.31	1.23,1.39	1.31	1.23,1.39
Study on BMI_0	0.14	-0.12,0.40	-0.01	-0.26,0.25
Study on BMI_{slope}	-0.05	-0.08,-0.02	0.02	-0.02,0.06
Study on FI_0	2.34	1.76,2.91	0.78	0.03,1.54
Study on FI_{slope}	-0.40	-0.54,-0.25	0.01	-0.22,0.23
Twin pair parameters (σ^2)				
Variance BMI_0	6.66	5.00,8.32	6.47	4.99,7.96
Covariance BMI_0 & BMI_{slope}	-0.51	-0.76,-0.27	-0.13	-0.38,0.11
Variance BMI_{slope}	0.05	0.02,0.08	0.02	-0.002,0.03
Variance FI_0	22.07	11.84,32.3	11.43	3.95,18.9
Covariance FI_{slope} & FI_0	-3.73	-5.75,-1.72	-3.47	-5.76,-1.17
Variance FI_{slope}	0.65	0.24,1.06	5.07	2.72,7.43
Covariance BMI_0 & FI_0	3.14	0.42,5.87	2.31	-0.40,5.01

Covariance BMI_0 & FI_{slope}	-0.44	-0.96,0.07	-5.56	-7.19,-3.94
Covariance BMI_{slope} & FI_0	-0.38	-0.69,-0.08	0.28	-0.09,0.65
Covariance BMI_{slope} & FI_{slope}	0.06	0.003,0.12	0.06	-0.16,0.28

Estimates and 95% confidence intervals were derived from dual change score models with and without bivariate coupling parameters. The models were adjusted for sex, smoking status, study, and twin-relatedness. The bivariate dual change score model with the best fit was a full coupling model, which included bidirectional coupling, with one coupling parameter linking BMI to FI change and one coupling parameter linking FI to BMI change. β_{BMI} denotes proportional change of BMI, β_{FI} denotes proportional change of FI, $\gamma_{BMI \rightarrow \Delta FI}$ denotes coupling parameters from BMI to FI change, $\gamma_{FI \rightarrow \Delta BMI}$ denotes coupling parameters from FI to BMI change.

Abbreviations: BMI – body mass index, FI – frailty index, CI – confidence intervals

Table S10: Estimates from dual change score models of BMI and functional aging index including variances and covariances

	No coupling		FAI-> BMI coupling model	
	**Estimate**	**95% CI**	**Estimate**	**95% CI**
Mean BMI intercept (μBMI_0)	26.95	26.37,27.52	26.10	25.47,26.74
Mean BMI slope (μBMI_{slope})	-1.77	-2.67,-0.87	-0.69	-1.57,0.19
Mean FAI intercept (μFAI_0)	35.99	33.85,38.13	36.49	34.54,38.43
Mean FAI slope(μFAI_{slope})	-2.23	-3.47,-0.99	-2.35	-3.53,-1.17
Proportional change parameters (β)				
β_{BMI}	0.06	0.03,0.10	0.05	0.02,0.08
β_{FAI}	0.09	0.07,0.11	0.09	0.07,0.11
Coupling parameters (γ)				
$\gamma_{FAI \rightarrow \Delta BMI}$			-0.02	-0.02,-0.01
Variances and covariances (σ^2)				
Variance BMI_0	9.74	8.16,11.32	9.86	8.22,11.5
Covariance BMI_0 & BMI_{slope}	-0.81	-1.14,-0.48	-0.75	-1.06,-0.43
Variance BMI_{slope}	0.09	0.04,0.13	0.09	0.05,0.13
Variance residual BMI	1.54	1.46,1.61	1.51	1.43,1.58
Variance FAI_0	42.18	30.17,54.2	41.16	28.75,53.57
Covariance FAI_{slope} & FI_0	-4.34	-6.33,-2.36	-4.10	-6.12,-2.08
Variance FAI_{slope}	0.62	0.31,0.94	0.56	0.25,0.88
Variance residual FAI	33.47	31.56,35.38	33.80	31.87,35.73
Covariance residuals BMI & FAI	-0.39	-0.69,-0.09	-0.36	-0.67,-0.05
Covariance BMI_0 & FAI_0	-0.21	-2.43,2.01	-1.03	-3.30,1.24
Covariance BMI_0 & FAI_{slope}	0.07	-0.3,0.44	0.16	-0.22,0.55
Covariance FAI_0 & BMI_{slope}	0.03	-0.26,0.32	0.69	0.31,1.06
Covariance FAI_{slope} & BMI_{slope}	-0.02	-0.07,0.02	-0.07	-0.12,-0.01
Parameters of covariates				
Mean sex	0.59	0.57,0.62	0.59	0.57,0.62
Variance sex	0.24	0.23,0.26	0.24	0.23,0.26
Sex on BMI_0	-0.26	-0.83,0.31	-0.30	-0.86,0.25

Sex on BMI_{slope}	-0.003	-0.07,0.07	0.07	-0.001,0.14
Sex on FAI_0	4.57	3.09,6.05	4.38	2.91,5.85
Sex on FAI_{slope}	-0.48	-0.71,-0.25	-0.45	-0.69,-0.21
Mean smoking	0.49	0.46,0.51	0.49	0.46,0.51
Variance smoking	0.25	0.23,0.27	0.25	0.23,0.27
Smoking on BMI_0	-0.61	-1.14,-0.08	-0.57	-1.09,-0.04
Smoking on BMI_{slope}	0.02	-0.04,0.09	0.02	-0.04,0.09
Smoking on FAI_0	0.73	-0.70,2.16	0.40	-0.97,1.78
Smoking on FAI_{slope}	-0.01	-0.22,0.19	0.04	-0.16,0.25
Mean study	-0.22	-0.28,-0.17	-0.22	-0.28,-0.17
Variance study	1.31	1.23,1.39	1.31	1.23,1.39
Study on BMI_0	0.14	-0.13,0.41	-0.21	-0.49,0.07
Study on BMI_{slope}	-0.05	-0.08,-0.02	-0.05	-0.08,-0.02
Study on FI_0	-3.10	-3.91,-2.29	-2.91	-3.71,-2.12
Study on FI_{slope}	0.27	0.17,0.37	0.25	0.15,0.34
Twin pair parameters (σ^2)				
Variance BMI_0	6.77	5.09,8.44	7.12	5.34,8.90
Covariance BMI_0 & BMI_{slope}	-0.54	-0.78,-0.29	-0.53	-0.78,-0.27
Variance BMI_{slope}	0.05	0.02,0.08	0.05	0.02,0.08
Variance FAI_0	15.15	6.58,23.73	15.65	5.59,25.71
Covariance FAI_{slope} & FI_0	-1.18	-2.51,0.15	-1.30	-2.87,0.27
Variance FAI_{slope}	0.08	-0.14,0.30	0.11	-0.14,0.35
Covariance BMI_0 & FAI_0	-0.17	-2.44,2.1	-0.94	-2.70,0.83
Covariance BMI_0 & FAI_{slope}	0.01	-0.34,0.35	0.10	-0.20,0.39
Covariance FAI_0 & BMI_{slope}	-0.12	-0.41,0.16	0.23	-0.08,0.53
Covariance FAI_{slope} & BMI_{slope}	0.02	-0.02,0.07	-0.01	-0.05,0.04

Estimates and 95% confidence intervals were derived from dual change score models adjusted for sex, smoking status, study, and twin-relatedness. The bivariate dual change score model with the best fit was a unidirectional model, with one coupling parameter linking FAI to BMI change. β_{BMI} denotes proportional change of BMI, β_{FAI} denotes proportional change of FAI, $\gamma_{BMI \rightarrow \Delta FAI}$ denotes coupling parameters from BMI to FAI change, $\gamma_{FAI \rightarrow \Delta BMI}$ denotes coupling parameters from FAI to BMI change.

Abbreviations: BMI – body mass index, CI – confidence intervals, FAI – functional aging index
